# Challenge of material haemocompatibility for microfluidic blood-contacting applications

**DOI:** 10.3389/fbioe.2023.1249753

**Published:** 2023-08-17

**Authors:** Gwenyth Newman, Audrey Leclerc, William Arditi, Silvia Tea Calzuola, Thomas Feaugas, Emmanuel Roy, Cécile M. Perrault, Constance Porrini, Mikhael Bechelany

**Affiliations:** ^1^ Department of Medicine and Surgery, Università degli Studi di Milano-Bicocca, Milan, Italy; ^2^ Eden Tech, Paris, France; ^3^ Institut Européen des Membranes, IEM, UMR 5635, Univ Montpellier, ENSCM, Centre National de la Recherche Scientifique (CNRS), Place Eugène Bataillon, Montpellier, France; ^4^ École Nationale Supérieure des Ingénieurs en Arts Chimiques et Technologiques, Université de Toulouse, Toulouse, France; ^5^ Centrale Supélec, Gif-sur-Yvette, France; ^6^ UMR7648—LadHyx, Ecole Polytechnique, Palaiseau, France; ^7^ Gulf University for Science and Technology (GUST), Mubarak Al-Abdullah, Kuwait

**Keywords:** haemocompatibility, microfluidics, surface modification, coating, medical devices, thrombosis, biomaterials

## Abstract

Biological applications of microfluidics technology is beginning to expand beyond the original focus of diagnostics, analytics and organ-on-chip devices. There is a growing interest in the development of microfluidic devices for therapeutic treatments, such as extra-corporeal haemodialysis and oxygenation. However, the great potential in this area comes with great challenges. Haemocompatibility of materials has long been a concern for blood-contacting medical devices, and microfluidic devices are no exception. The small channel size, high surface area to volume ratio and dynamic conditions integral to microchannels contribute to the blood-material interactions. This review will begin by describing features of microfluidic technology with a focus on blood-contacting applications. Material haemocompatibility will be discussed in the context of interactions with blood components, from the initial absorption of plasma proteins to the activation of cells and factors, and the contribution of these interactions to the coagulation cascade and thrombogenesis. Reference will be made to the testing requirements for medical devices in contact with blood, set out by International Standards in ISO 10993-4. Finally, we will review the techniques for improving microfluidic channel haemocompatibility through material surface modifications—including bioactive and biopassive coatings—and future directions.

## 1 Introduction

**FIGURE 1:**
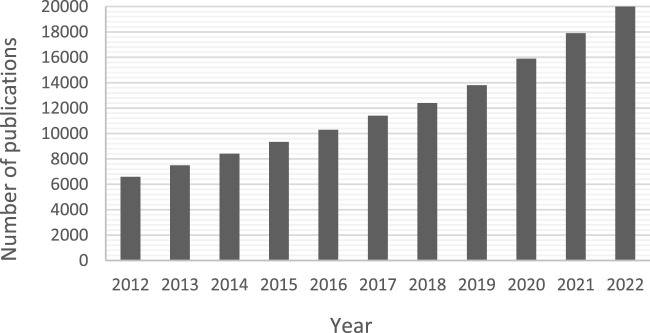
Number of publications (including patents) per year from 2012 until 2022 from searching Google Scholar with the term “microfluidics + blood −brain”. The results including the word “brain” were removed to exclude irrelevant organ-on-chip papers focussing on the blood-brain barrier.

Material haemocompatibility of blood-contacting medical devices poses a major challenge to their long-term implementation (Jaffer et al., 2015). The haemocompatibilty of a material is the evaluation of the effects experienced by blood when interfaced with said material. Interactions between blood components and biomaterials contributes heavily to the process of medical device-induced thrombosis, a process which is difficult to curb once initiated (Hilal et al., 2019). The application of coatings is a crucial step in protecting the blood from material-induced damage, however their performance can vary depending on the substrate material, device dimensions and method of application (Fotovvati et al., 2019). The incorporation of new technologies—Such as microfluidics—In medical devices introduces new requirements and challenges to this process. Therefore, although previous reviews exist on the modes of action of haemocompatible coatings, there remains a lack of publications detailing microfluidic-specific applications. The growing interest in microfluidics for blood related applications can be observed by the increasing papers and patents published in this research area over the last decade, shown in Figure 1.

### 1.1 Microfluidics for blood-contacting applications

Microfluidics technology is based on the different phenomena experienced by fluid manipulated in microscale channels when compared to classical fluid dynamics. Under these geometrical constraints the surface area of the channel is much greater compared to the volume of fluid flowing through the channel, resulting in a shift in the force balance and a characteristically laminar flow profile (Beebe et al., 2002). In laminar flow regimes viscous forces dominate and particles follow precise streamlines without mixing across adjacent fluid layers, and hence mass transport processes are dominated by diffusion. The resulting system has highly predictable characteristics based on convection-diffusion equations and Fick’s law, generating precise and tuneable gradients (Fan et al., 2018).

Microfluidics in the biomedical field has been dominated by diagnostics, cell and DNA analysis and organ-on-chip devices (Low et al., 2021). These platforms have the advantage of requiring less reagents, space and energy, minimising sample size. Therefore it may seem counter-intuitive for microfluidics to find use in high volume and high flow rate applications. Nonetheless, the precise control of diffusion gradients, short diffusion distances, and efficient fluidic transport in microfluidic channels have provided a window of opportunity for applications in another biomedical field: blood treatment. Research and development of microfluidic-based oxygenators and haemodialysers has grown in the past decade (Lee et al., 2008; Wei Hou et al., 2012; Potkay, 2014; Rochow et al., 2014; Kovach et al., 2015; D’Amico et al., 2017; Ausri et al., 2018; Yoon et al., 2019; Dabaghi et al., 2020; Luo et al., 2020; Gimbel et al., 2021; Lachaux et al., 2021; Santos et al., 2021).

A critical design consideration is haemocompatibility. In order to improve on current clinical devices, they must offer a less damageable experience for the blood over extended periods of time (from weeks to months). Microfluidic channels have an inherently high material surface-area to fluid volume ratio and therefore the blood-material interactions are even more pertinent. Furthermore, the small scale of microchannels means that even small thrombi can cause flow disruptions or blockage. This is therefore a key focus point to establish clinical feasibility of blood-contacting microfluidic medical devices, and a review of the literature in this field will support this research.

### 1.2 Common materials for microfluidic devices

Since the emergence of microfluidics for biomedical applications, the choice of device material—And related fabrication method—Has been a critical one. The most widely used materials for biomedical microfluidic devices are polymers, namely polydimethylsiloxane (PDMS) and thermoplastics, such as polystyrene (PS), polycarbonate (PC), cyclo-olefin copolymers (COC) and poly(methyl methacrylate) (PMMA). Multiple reviews have described the material properties in the context of microfluidic materials in depth (Tsao and DeVoe, 2009; Van Midwoud et al., 2012; Roy et al., 2016; Tsao, 2016; Gencturk et al., 2017; Voicu et al., 2017; Nielsen et al., 2020).

Briefly, although PDMS and thermoplastics—Particularly PS and PC, from which cell culture labware is made—Are acknowledged as biocompatible (Bélanger and Marois, 2001), this property is not evaluated in the same way as haemocompatibility (Picone et al., 2019). The definition of biocompatibility is the ability of a biomaterial to operate as intended without causing unwanted and inappropriate effects in the host (Williams, 2008). The focus of biocompatibility tests is generally in cell and tissue responses: viability, oxidative stress and enzymatic changes (Bélanger and Marois, 2001; Picone et al., 2019). A major downfall of PDMS and thermoplastics for blood-contacting applications is surface hydrophobicity (Mukhopadhyay, 2007; Tan et al., 2010; Van Midwoud et al., 2012; Gokaltun et al., 2017; Miranda et al., 2022) leading to adsorption of molecules to their surfaces and triggering coagulation pathways, as demonstrated in Figure 3. As well as absorbance concerns, PDMS is an unstable polymer with issues of unpolymerised oligomers leaching out of the material (Regehr et al., 2009; Berthier et al., 2012; Carter et al., 2020).

Gas permeability is an important consideration for blood-transporting channels to ensure blood gas levels remain between physiological limits (Beetham, 1982; Castro et al., 2022). PDMS has a high permeability to oxygen, with a coefficient of 600 Barrer (Evseev et al., 2019) or ∼2000–4000 μm^2^ s^−1^ (Berthier et al., 2012), a value much greater than the other polymers. Changes in gas distributions risks bubble formation, potentially resulting in cell membrane rupture, embolism, or disruption to flow profiles (Heo et al., 2007; Berthier et al., 2012).

In this paper we will discuss material haemocompatibility, from the initial contact between blood and biomaterial, through the mounting of the coagulation cascade and formation of thrombi. The International Standards for testing haemocompatibilty will be reviewed, in the context of microfluidics and the common materials used in the fabrication of these devices. Following from this, the practical challenges of applying haemocompatible surface treatments to microchannels and the state of the research of microchannel coatings will be discussed, with brief reference to commercially-available coatings. We will finish by predicting the future direction of haemocompatible treatments for microfluidic channels.

## 2 Material haemocompatibility

**FIGURE 2:**
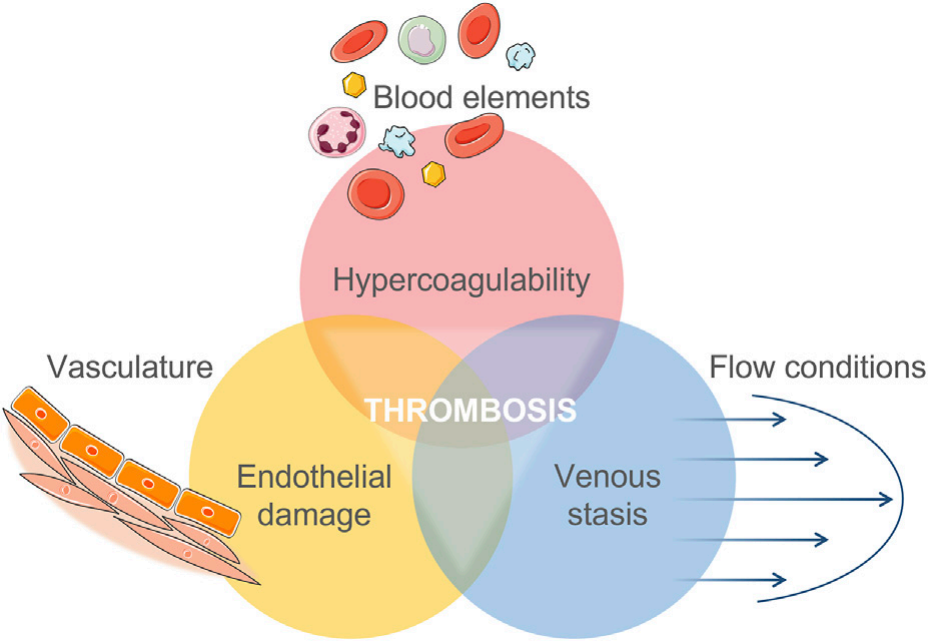
Virchow’s Triad of interacting factors which contribute to an increased risk of thrombosis. Adapted from Wolberg et al. (2012) combined with Virchow’s original translation.

Blood is a characteristically heterogenous fluid, comprised of plasma and cells. Cells—Red blood cells, white blood cells, and platelets—Constitute 45% of blood volume. The main role of platelets is in haemostasis, reducing loss of blood after vessel injury. Plasma is a complex mixture of water, fats, salts and a diverse proteome (Anderson and Anderson, 2002). The active nature of the blood means that interactions with foreign materials in the bloodstream can have far-reaching and detrimental effects. It is well-documented that blood coagulation is a complex, interrelated symphony of material, mechanical, and biological factors; first conceptualised by Virchow in the mid-19th century (Virchow, 1856; Kumar et al., 2010) and revisited throughout the years (Chung and Lip, 2003; Lowe, 2003; Wolberg et al., 2012). The Virchow Triad, represented in Figure 2, combines the original elements of Virchow’s theory, adapted according to Wolberg et al. (2012). This section will focus on the interaction between the material and blood elements.

### 2.1 Protein adsorption

**FIGURE 3:**
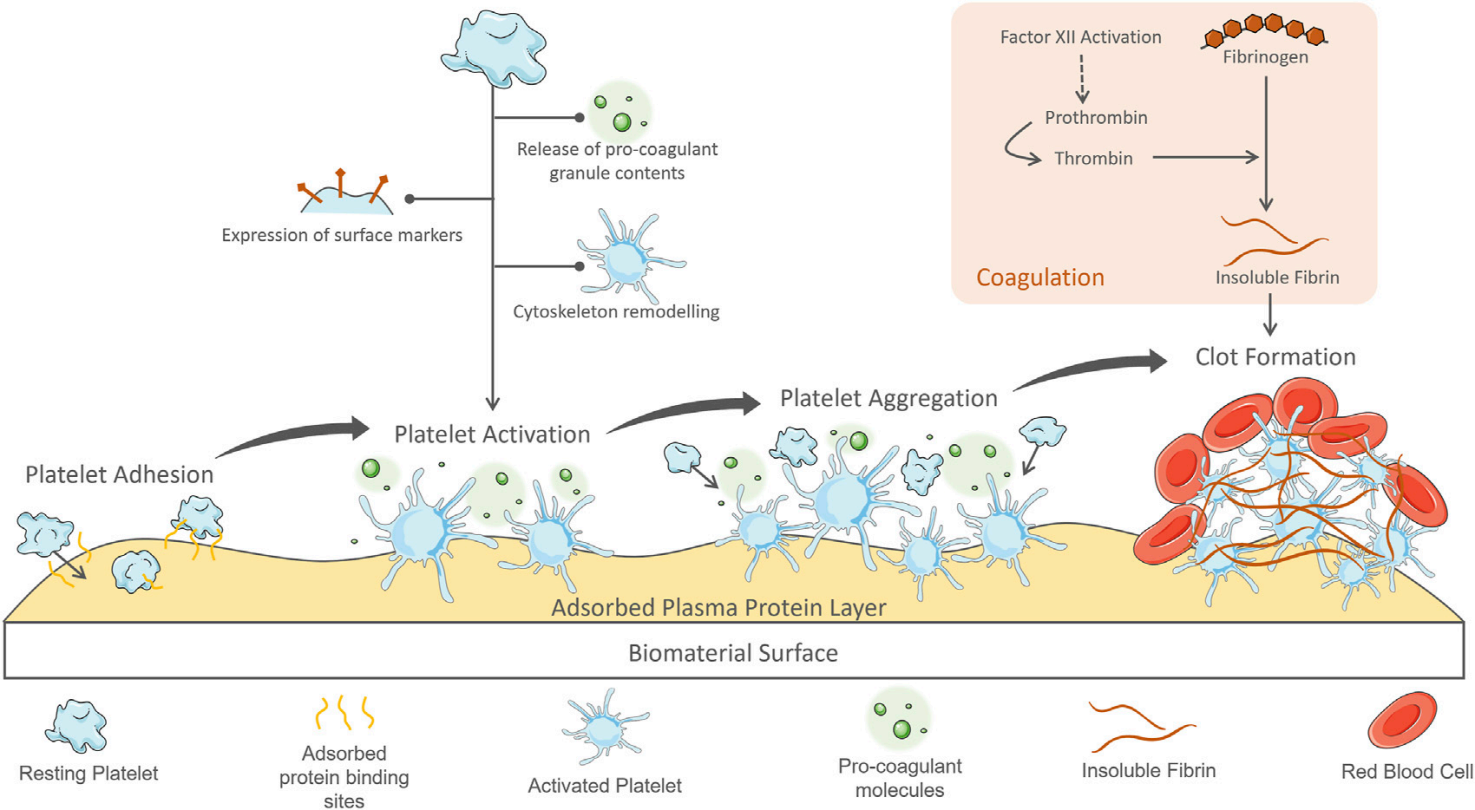
Process of plasma protein adsorption to a blood-contacting biomaterial surface and ensuing platelet activation and coagulation response leading to thrombus formation.

Immediately upon contact with a foreign material plasma proteins are adsorbed onto the surface, the initial step in the pathway which eventually results in thrombus formation (Figure 3). The composition of the protein layer depends on the biomaterial properties and individual protein concentration—Proteins with greater affinity will displace those less attracted in a process termed the Vroman effect (Simmonds et al., 2018). Mass spectrophotometry techniques have been employed to analyse these protein films and their development over time (Xia et al., 2002; Wagner and Castner, 2004). This method identifies molecules based on their mass-to-charge ratio, through ionisation of a surface by bombardment with electrodes, then collection and characterisation of these dispersed fragments. The adsorbed proteins form a monolayer of thickness 2–10 nm (Wilson et al., 2005; Jaffer et al., 2015). On adsorption to a surface, plasma proteins undergo a conformational change, allowing them to interact with coagulation factors and blood cells—Most notably platelets (Sivaraman and Latour, 2010; Zhang et al., 2017; Horbett, 2018). The interactions have been well-documented for the most prevalent, and problematic, proteins of the adsorbed layer: fibrinogen and albumin.

#### 2.1.1 Albumin

Albumin comprises about 55% of the proteome (Anderson and Anderson, 2002), and rapidly diffuses and is one of the first proteins adsorbed to surfaces, however it soon becomes displaced by the Vroman effect, being replaced by proteins with a higher affinity for the surface (Leonard and Vroman, 1992; Wilson et al., 2005). Albumin does not contain binding sites for platelets and is generally considered to be inert, which promotes its use as an option in biopassive coatings, as will be discussed later (Hylton et al., 2005). Of these higher-affinity proteins, fibrinogen has the most significant effects.

#### 2.1.2 Fibrinogen

Fibrinogen, a major coagulation factor in plasma, plays a critical role in material contact-initiated blood activation. Under physiological conditions, soluble fibrinogen circulates in the blood in an inactive form (Hylton et al., 2005) and only on vessel injury does it reveal its coagulation properties. Following initiation, subsequent factors of the coagulation cascade are activated, leading to the generation of thrombin (Wolberg et al., 2012). Thrombin is an enzyme which catalyses the conversion of fibrinogen dissolved in plasma into fibrin fibres. These polymerise to form dense, branched networks which provide mechanical support to thrombi encompassing cells and clotting factors. In the physiological environment, these aggregates form at damage site to reduce bleeding and promote healing (Kalathottukaren and Kizhakkedathu, 2018). However, during material contact-initiated thrombosis this coagulation process contributes detrimentally. Firstly, fibrinogen is a surfactant (Horbett, 2018). It is a large molecule with many and varied side chains providing regions for bonding to biomaterial surfaces. This results in a rapid and wide-spread surface adsorption, only enhanced by conformational unfolding which increases available protein-surface interactions. This conformational change exposes platelet binding sites on fibrinogen which tethers circulating platelets to the surface—It is not the amount of adsorbed protein, but instead the shape change which mediates platelet adhesion (Sivaraman and Latour, 2010). Fibrinogen is over-represented in the adsorbed protein layer compared to its low concentration in plasma (5% of total protein content). Furthermore, fibrinogen can promote platelet adhesion even at very low concentrations on a material surface. Park et al. first identified the minimum adsorbed concentration of fibrinogen required for platelet activation on a glass-based substrate to be 20 ng/cm^2^, or between 2%—15% surface coverage (Park et al., 1991). The maximal platelet adhesion was reported by Tsai et al. to occur with 5–10 ng/cm^2^ of adsorbed fibrinogen to a polystyrene surface (Tsai et al., 1999). These values are extremely low considering that fibrinogen has been shown to adsorb from plasma to polymer surfaces between 100—200 ng/cm^2^ (Horbett, 2013). Adsorption of fibrinogen can be used as a measure of material haemocompatibility.

### 2.2 Platelet Activation

Platelets adhered to the adsorbed protein layer on biomaterial surfaces exhibit similar pro-coagulant behaviour as during platelet plug formation following vessel injury. The extrinsic coagulation pathway initiates at sites of vessel damage where tissue factor, collagen and other proteins usually contained below the vascular endothelium have become exposed to the blood (Thomas, 2019; Neubauer and Zieger, 2021). These proteins bind and activate clotting factors and circulating platelets, triggering the coagulation cascade. In normal physiology, adhered platelets must withstand the shear forces exerted by flowing blood to maintain their position at the site of vessel damage (Kamath et al., 2001). To achieve this, platelets utilise many binding points specific for extracellular matrix proteins. In the case of biomaterial contact, the adsorbed protein layer provides these docking points for platelets, leading to a similar stubborn adhesion. Once adhered, the platelets become activated, undergoing a morphological change as occurs in the formation of the platelet plug during haemostasis (Kalathottukaren and Kizhakkedathu, 2018; Thomas, 2019). Cytoskeleton remodelling dramatically alters the platelet shape to facilitate their haemostatic functions of spreading and aggregation. Through the polymerisation of actin filaments, the resting disc shape spreads with finger-like filopodial extensions, reaching up to 420% of its resting surface area (Daimon and Gotoh, 1982; Thomas, 2019). This extended morphology catches other circulating blood cells. The resulting structure rapidly covers the surface forming aggregates structurally supported by fibrin.

Another important feature of platelets is their α-granules, dense granules and lysosomes, which are rapidly released into the extracellular space on activation (Kamath et al., 2001; Blair and Flaumenhaft, 2009; Flaumenhaft and Sharda, 2018; Thomas, 2019). Granule contents include coagulants, adhesion proteins, growth factors, bioactive amines, immune mediators, chemokines, proinflammatory molecules and angiogenic factors (Flaumenhaft and Sharda, 2018). Activated platelet surface markers, such as P-selectin, initiate interactions with other cells and the material surface (Blair and Flaumenhaft, 2009). Adhesion proteins (such as fibrinogen and von Willebrand Factor) released from granules enhance these interactions. Released prothrombin contributes to the coagulation cascade in the formation of insoluble fibrin. High concentrations of pro-inflammatory chemokines are released from granules, propagating further platelet activation in a positive-feedback loop (Blair and Flaumenhaft, 2009; Thomas, 2019; Labarrere et al., 2020).

These responses of the blood on contact with biomaterials can be markers for testing of material haemocompatibility.

### 2.3 Evaluating material haemocompatibility with ISO 10993-4

The common polymers used for microfluidics in biomedical applications, their biocompatibility, haemocompatibility and gas permeability are summarised in Table 1.

**TABLE 1:** Features of common polymers used in the fabrication of microfluidic devices for biomedical applications.

Polymer	Biocompatibility (according to ISO 10993 or USP class VI)	Hemocompatibility	Gas permeability to O2 (Barrer)
PDMS	Biocompatible Victor et al. (2019)	Not haemocompatible Van Oeveren et al. (2002)	600 Potkay (2014)
PS	Biocompatible Vesel et al. (2011)	Limited data available	4.75 ± 0.2 Compton et al. (2010)
PC	Biocompatible Gómez-Gras et al. (2021)	Not haemocompatible Krishnamoorthi et al. (2023)	1.68 Lai et al. (1993)
COC	Biocompatible Bernard et al. (2017)	Limited data available	0.72–2.39 Hu et al. (2006)
PMMA	Biocompatible Pituru et al. (2020), Díez-Pascual (2022)	Limited data available	0.26 Borandeh et al. (2019)

Regarding the haemocompatibility of polymers, there is lack of published data to confirm their status. In addition, even when some companies develop regulated products, they do not always disclose all the tests carried out to obtain their certification. This lack of transparency reveals the need for further research in this field, in order to produce a robust evaluation of polymer haemocompatibility. Investigations into material haemocompatibility have fallen behind those for biocompatibility, perhaps revealing a complacency in medical device research to accept biocompatibility approval as sufficient for blood-contacting applications. However, specific haemocompatibility tests are required—Listed in International Standard (ISO) 10993-4.

ISO 10993 details the biological evaluation of medical devices, with part 4 dedicated to the selection of tests for interactions with blood. The types of devices discussed in this review fall into the categories of external communicating devices directly contacting circulating blood. The standard defines a haemocompatible device or material as: “*able to come into contact with blood without any appreciable clinically-significant adverse reactions*”. The listed test categories check for these adverse reactions: haematology, haemolysis, platelet response, thrombosis, coagulation, and immune response. It is critical to carry out these tests under specific conditions in order to achieve relevant results.

#### 2.3.1 Test operating conditions

According to the standard, the pre-treatment and analysis of blood samples are critical for correct interpretation of *in vitro* testing. Haematocrit, anticoagulant treatment, sample origins, sample preparation and storage, aeration, pH, temperature, contact surface area to sample volume ratio are listed as important variables to consider. Under dynamic test conditions, the list of variables extends to include flow rate, wall shear rate and pressure drop. The delay between blood sample collection and testing should be limited as much as possible (less than 4 h) in order to reduce non-physiological changes in blood properties.

These tests are simulated under *in vitro* operating conditions, replicating the blood-device interaction as closely as possible. Contact time, exposure ratio (surface area of material or device to blood volume ratio), temperature, sterility, and dynamic conditions (flow rate, shear rate, and pressure drop) should be specified to achieve this. Since the conditions in microfluidic channels are very specific, this is a crucial consideration when testing these devices.

#### 2.3.2 Haematology

A Complete Blood Count (CBC) assesses the haematology of a blood sample. This is a standard clinical assessment carried out in medical laboratories. It returns the cell population counts, haematocrit and haemoglobin content from a blood sample via a series of tests run through a haematology analyser (Buttarello and Plebani, 2008). When compared to standard or control values this test can identify the loss of cells, either through retention on a material surface or destruction.

#### 2.3.3 Haemolysis

Haemolysis is the rupture of the erythrocyte cell membrane. This can be biochemically-induced due to contact with the device material or changes in blood pH, temperature or nutritional factor content. Mechanical effects such as non-physiological shear stresses, turbulent flow paths or osmotic pressure differentials can induce haemolysis (Köhne, 2020). When the erythrocyte membrane ruptures haemoglobin is released into the surrounding plasma and can be detected. The level is reported as the percentage of free haemoglobin to the total haemoglobin concentration (Shin et al., 2021). Clinically, haemolysis is a concern due the loss of erythrocytes which can result in anaemia. Excessive levels of haemoglobin can induce toxic effects on organs and may also contribute to thrombotic processes. ISO 10993-4 states that it is not possible to assign a general value for pass or fail of haemolysis assessment, the situation is more nuanced. The duration of exposure to the device, consistency of haemolysis throughout its use, and comparison to alternative treatments must be considered in risk-benefit analysis.

#### 2.3.4 Platelet status

The attachment of platelets to the surfaces of medical devices can be quantified by various techniques. Running a platelet count before and after interaction with the device quantifies total reduction in platelets. Alternatively, direct counting of adhered cells per unit area can be completed by scanning electron microscopy of the surface. This method also allows for the cell morphology to be assessed. Platelets can be labelled prior to testing and the intensity of signal (fluorescence or radiation) can be used to determine the surface-bound cells (Weber et al., 2018; Braune et al., 2019). A similar method indirectly quantifies the platelet adhesion through apoptosis assays, such as LDH (lactate dehydrogenase). However, it must be noted that for these indirect tests platelet-rich plasma should be used rather than whole blood, otherwise the cell type is not certain.

Flow cytometry analysis is the most common method for determining the active state of a platelet population through detection of the membrane markers P-selectin (CD62) or CD42b which are expressed only when activated (Braune et al., 2019). Assays can be carried out to quantify soluble granule contents released by activated platelets, such as the amount of Platelet Factor 4 (PF4) or β-thromboglobulin (Braune et al., 2019). Alternative methods include qualitative investigation of platelet morphology with scanning electron microscopy.

#### 2.3.5 Coagulation

The coagulation status of blood after contact with materials can be investigated through the generation of thrombin and fibrin, the major proteins of the pathway. This can be achieved by conducting ELISA for thrombin-antithrombin complex and fibrinopeptide A respectively (Braune et al., 2019).

#### 2.3.6 Immune response

According to ISO 10993-4 the immune response takes into account the complement system and leukocyte functions (Weber et al., 2018; Braune et al., 2019). The number of leukocytes in a blood sample is reported in the CBC, and their adhesion to the material surface can be quantified by the same approach as for platelets. The active state of leukocytes is confirmed through the detection of PMN elastase, an enzyme secreted by activated leukocytes as part of the inflammatory response (Lee and Downey, 2001). The complement system activity is determined by the presence of proteins which are activated in a cascade during an immune response (Dunkelberger and Song, 2010). Since there are different pathways by which the complement system can be triggered, either protein C3a—Which is common to all pathways—Or the terminal product SC5b-9 is investigated. Their detection can determine if the complement system is active and the immune response has been mounted.

#### 2.3.7 Limitations of haemocompatibility tests

Due to the micron scale dimensions, microfluidic channels are less tolerant to blockages and even minimal surface deposition can dramatically affect the hydrodynamic forces and therefore the device operation. There is a need to develop microfluidic-specific tests, or adapt the current ISO test recommendations to the operation of these devices, in order to ensure appropriately haemocompatible devices. The testing method outlined in this standard is only sufficient in investigating blood-biomaterial interaction and are limited in their ability to predict a device’s holistic effect on the blood.

When considering erythrocytes, haemolysis is the standard ISO test to confirm material haemocompatibility, however it represents a high level of destruction to red blood cells which occurs in extreme conditions (Gusenbauer et al., 2018). The measurement does not consider the damages imposed prior to membrane rupture: pore formation, tether formation, changes to cell shape and membrane flexibility (Grigioni et al., 2005). This sublethal damage can result in early removal of cells from the bloodstream and consequently low haematocrit can result in anaemia (Olia et al., 2016). Although not included in ISO 10993-4, these effects can be identified by investigating red blood cell deformability through micropipette aspiration, viscometry or optical methods (Olia et al., 2016). The inclusion of sublethal damage tests would improve on current haemolysis detection techniques.

In extracorporeal devices, the entire blood volume will pass through before re-entering the vasculature and continuing circulation in the body. Therefore the damage to the blood will not be equivalent to the same volume of blood being repeatedly circulated through the device. However, it must also be noted that these devices are intended for long term use and therefore any deposition of blood products on the channel surfaces will contribute to a build-up over time.

The flow element part of Virchow’s triad provides another avenue for design optimisation and testing through computational modelling. This is particularly interesting in microfluidic devices where the flow conditions are highly specific, predictable, and can be manipulated with close precision. The methods by which this can be achieved have been summarised in a recent review (Feaugas et al., 2023).

## 3 Blood-protective coatings

Coating of biomedical materials is standard practice for devices in contact with blood: from basic tubing to heart valves, stents and ECMO circuits. However, this remains a hurdle for new devices seeking clinical approval and development of haemocompatible coatings is an area of active research (Hedayati et al., 2019). This section will cover the unique challenges when coating microfluidic channels, pre-treatment of surfaces with plasma exposure, the haemocompatible coatings which have been applied to microchannels, and finally commercially-available coatings.

### 3.1 Challenges in coating microchannels

**FIGURE 4:**
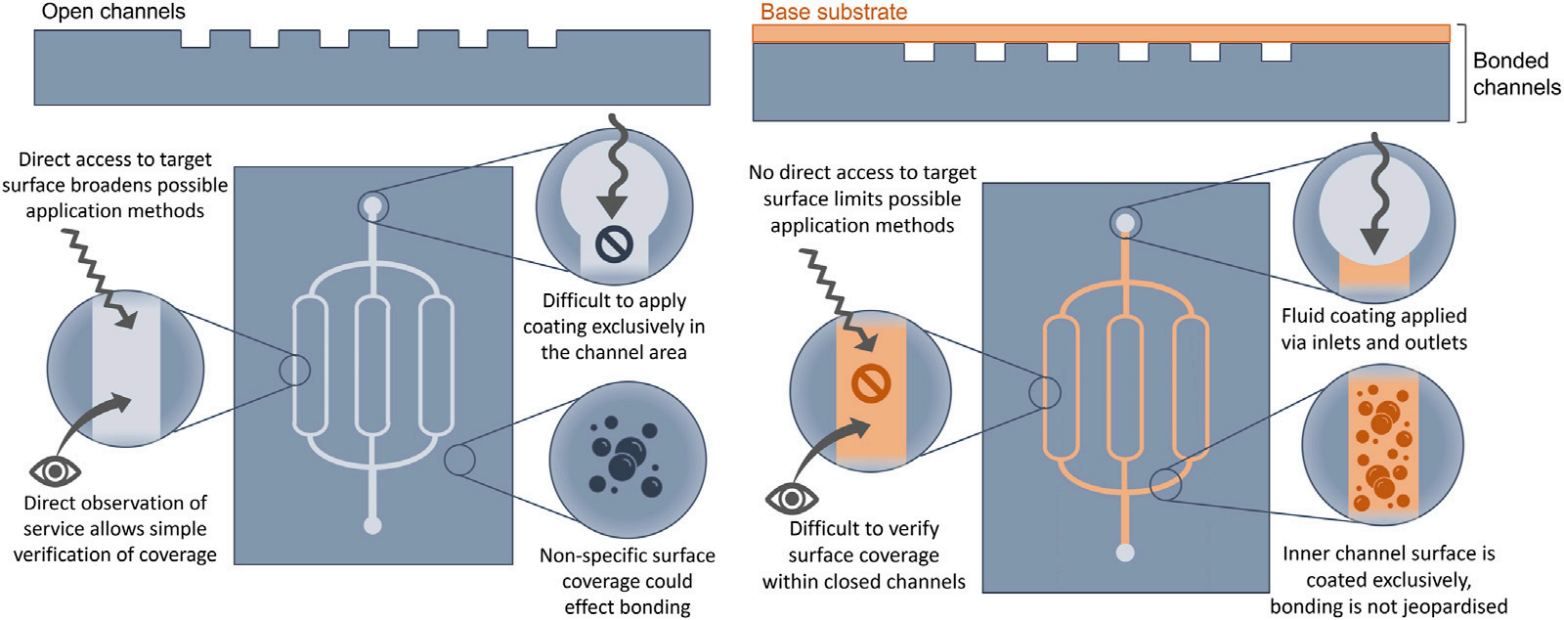
Differing coating applications to open (left side) and sealed (right side) microchannels, with practical features of each: benefits and challenges.

Typical well-established surface coating methods include dip-coating, spray-coating and physical/chemical vapour deposition (Fotovvati et al., 2019). Microfluidic channels pose unique challenges which may deem these methods inappropriate, or else require substantial adaptions. Considering that most microfluidic chips are fabricated by the ‘sandwich’ method of creating open channels and then bonding them to a base substrate to seal (Convery and Gadegaard, 2019), coatings can be applied before or after this bonding step—With each approach bringing its own benefits and challenges, summarised in Figure 4.

Coating of open channels (prior to bonding) provides direct access to the target surface which is more likely to support the use of traditional techniques and equipment, and broadens the viable methods. Furthermore, verification of coating coverage can be carried out more reliably on an exposed surface where fluorescent and microscopy techniques can be employed. However, many traditional coating methods cover the material surface totally and non-specifically, but in microfluidics it is only the inner channel surface that requires coverage. Coatings applied to the substrate area around the channels is likely to alter bonding properties due to changed surface chemistry. Since sealing is already a major challenge further complications should be avoided (Tsao, 2016). In parallel, bonding often requires surface pre-treatment (Tsao and DeVoe, 2009; Sivakumar and Lee, 2020) which may interfere with the coating. To apply the coating only to the inner channel area requires microscale precision to deposit only on target areas.

To overcome this, microchannels may be coated after sealing. This approach, however, would require a fluidic coating method which can enter the channels via the inlets and outlets. As these devices have controlled operational conditions (such as hydraulic pressure and resistance) and the coatings often have their own requirements (such as exposure time and mode of reaction) and fluidic qualities (such as viscosity and chemical composition) it can be difficult to achieve a reliable coverage. Many surface treatment methods require direct interaction with the surface and are incompatible with a closed channel approach, such as plasma treatment (Sundriyal et al., 2020). Additionally, evaluation of coverage is difficult to achieve without opening the channels.

### 3.2 Plasma treatment

An initial step in polymer surface treatment is to increase the hydrophilicity. Plasma treatment (with either oxygen or air) can achieve this by degrading contaminants on the surface and introducing oxygen-containing functional groups (Lai et al., 2006). Plasma treatment directs high energy ions towards the surface, breaking organic bonds which are free to react with oxygen species in the gas to result in a negatively-charged surface with increased hydrophilicity (Yu et al., 2015; Iqbal et al., 2019; Primc, 2020; Jiang et al., 2022). However this method alone does not provide sufficient haemocompatibility (Recek et al., 2014), mainly due to hydrophobic recovery of the material or incomplete coverage of oxygen-containing groups (Mukhopadhyay, 2007; Zhou et al., 2012; Zahid et al., 2017). Therefore it is often used in combination with other haemocompatible coatings. The free radicals on the material surface after plasma treatment can be reacted with other molecules to cover the surface (Sharma et al., 2007; Shakeri et al., 2019; Özgüzar et al., 2022). Since plasma treatment is already a crucial step in microfluidic device fabrication, it positions this as a convenient and accessible option for surface pre-treatment. A plasma machine is a fundamental piece of equipment in microfabrication laboratories and its effects can be utilised simultaneously for bonding, cleaning and surface treatment purposes (Zhou et al., 2012; Sundriyal et al., 2020; Hassanpour-Tamrin et al., 2021).

#### 3.2.1 Polymer stability

As previously mentioned, PDMS stability is a major concern when used in biomedical applications. Low molecular weight chains migrate from the interior of the PDMS to the surface by diffusion (Bodas and Khan-Malek, 2007), resulting in a return of the material’s original hydrophobic properties (Lillehoj and Ho, 2010; Long et al., 2017; Trantidou et al., 2017). This can occur rapidly, from hours to days after surface modification, questioning its suitability for longer term biomedical use. It has been reported that after surface hydrophilisation by plasma treatment, the hydrophobic surface returns after 30 min to 2 h—Depending on the plasma parameters used (Stauffer, 2014; Long et al., 2017). As already discussed, PEGylation of the PDMS surface improves its hydrophilic stability (Lai and Chung, 2020), but the polymer can be further stabilised through modification of its structure with PEG. The addition of PEG to uncured PDMS results in a stable surface hydrophilicity for up to 20 months (Gökaltun et al., 2019).

COC polymers also have stability issues. After plasma oxygenation, they are only stable for a few hours before returning to their hydrophobic character due to the reorganization of the polymer or contamination by air (Lee et al., 2015).

PC, a polymer, that is, neither strongly hydrophilic nor hydrophobic, is also one of those polymers whose hydrophilic nature can be improved by treatments, but not permanently. On the other hand, it is possible to stabilize it for at least 1 year by modifying its surface by reaction with branched polyethyleneimine as well as with a poly(ethylene-alt-maleic anhydride) anhydride and then by the hydrolysis of the anhydride groups (Jankowski and Garstecki, 2016). The PC surface thus contains a large amount of carboxyl groups.

Increasing the power intensity of the oxygen plasma treatment improves the stability time of PC and PS. Following treatment with a self-polarization voltage between 480 and 600 V, the polymers are still hydrophilic even after 6 months of storage (Larsson and Dérand, 2002).

The stability of PMMA surface hydrophilicity is improved this time by plasma treatment followed by fixation of polyvinylalcohol (PVA). In dry storage conditions, the PMMA could keep a contact angle between 10° and 20° for at least 1 month post-treatment. Tests were only conducted after 30 days so the stability beyond this period is still to be investigated (Yu et al., 2015).

### 3.3 Types of coating

#### 3.3.1 Heparin

Bioactive haemocompatible coatings harness the pharmacological properties of anticoagulant molecules to intervene with thrombotic pathways. Heparin is a very common and widely used anti-coagulant drug in clinical medicine and is the gold-standard coating of medical devices, from general tubing to vascular stents and extracorporeal devices (Olsson and Larm, 1991; Biran and Pond, 2017; Jaffer and Weitz, 2019). It is a molecule based on heparan sulphate expressed on the surface of endothelial cells where it binds with anti-thrombin to counteract thrombin’s platelet stimulation (Kuchinka et al., 2021). The catalytic mode of action of heparin is the same, it binds anti-thrombin at a specific active site (Casu, 1985). It is therefore crucial that this active penta-saccharide sequence found at the end of the heparin molecule is freely available for bonding with anti-thrombin. This adds further challenge to the method by which heparin can be attached to a substrate. Heparin has been immobilised on polymer surfaces by different methods, requiring initial activation or surface functionalisation which includes polymer brush spacers, protein layers or amination (Magoshi and Matsuda, 2002; Michanetzis et al., 2003; Olander et al., 2003; Chen et al., 2005; Linhardt et al., 2008; Du et al., 2011; Kolar et al., 2015; Biran and Pond, 2017; Özgüzar et al., 2022). These will be covered in the following sections.

Heparin treatment, however, does have drawbacks. The most severe is heparin-induced thrombocytopenia (HIT), a condition whereby the patient’s immune cells are triggered to activate platelets on contact with heparin (Hilal et al., 2019). This has a rapid, pro-coagulant effect on the blood, forming clots which can have severe effects. HIT occurs in approximately 2.5% of patients, with a greater risk during recurring heparin treatments (Maul et al., 2016)—Such as during extra-corporeal therapies. It must also be noted that heparin operates by thinning the blood, therefore there is the associated risk of uncontrolled bleeding.

#### 3.3.2 Albumin

Biopassive coatings aim to eliminate protein adsorption to the surface from first contact with blood, stopping initiation of coagulation cascade. They act through “stealth” techniques—Rendering the material surface inert. One such method is blocking by albumin (Sweryda-Krawiec et al., 2004; Hylton et al., 2005; Yamazoe et al., 2010; Zhang et al., 2013; Krajewski et al., 2015; Park et al., 2017). Albumin is the most abundant plasma protein and its structure does not possess platelet-binding sites. Intentional saturation of the material surface with albumin creates a protective layer which blocks binding of other proteins and the ensuing activation of coagulation pathways (Hylton et al., 2005). It can be applied as a solution through sealed channels making it well-suited for coating of microfluidic networks (Schrott et al., 2009; Convert et al., 2012; Azizipour et al., 2022). Furthermore, surface pre-treatment is not required for this method of coating (Guha Thakurta and Subramanian, 2011; Park et al., 2017). However, albumin can denature, dissolve or be displaced by fibrinogen over time, triggering the pathways described earlier (Amiji et al., 1992; Zelzer et al., 2012; Buddhadasa et al., 2018; Hasan et al., 2018). Albumin alone is considered better suited to short term applications, but can be used in combination with other strategies to improve its durability.

#### 3.3.3 Polymer brushes

Polymer layers can be added to surfaces to limit protein adsorption, often referred to as polymer brushes because of their adhesion to the surface at one end of the chain (Buhl et al., 2020). Polymers based on the ethylene glycol unit, such as polyethylene glycol (PEG) and Poly (ethylene oxide) (PEO) (Leckband et al., 1999), are commonly bonded to surfaces as coatings to prevent non-specific protein fouling (Israelachvili, 1997; Wei et al., 2014; Fischer et al., 2018). This process is often referred to as PEGylation and has been achieved on the channels of PDMS microfluidic devices by plasma pre-treatment (Sharma et al., 2007) or integration as a copolymer additive (Gökaltun et al., 2019). PEGylated PDMS microchannels exhibit improved haemocompatibility—With reduced fibrinogen adsorption, platelet adhesion and platelet activation when compared to bare surfaces (Yeh et al., 2012; Kovach et al., 2014; Plegue et al., 2018; Gökaltun et al., 2019). This is accredited to the hydrophilic properties of PEG-based materials, whereby hydrogen bonds to the surface, creating a steric repulsion and a physical water barrier to reduce surface fouling (Chen et al., 2010; Long et al., 2017; Thon et al., 2017; Plegue et al., 2018).

Polymer brushes have also found application as spacers through immobilisation of specific molecules to the free end of the polymer chain, exposing it to the contacting blood (Wong and Ho, 2009; Wei et al., 2014; Kolar et al., 2015). PEG has reactive terminal hydroxyl groups which provide a platform for grafting of bioactive molecules or with other polymers to improve the coating properties. When used in this way, polymer brushes provide a secure grafting to the material surface and an end-point which can interact with the desired molecule. The bio-inspired polydopamine (PDAM) has gained interest for having great potential as a method of surface functionalisation by this approach (Liu et al., 2014; Madhurakkat Perikamana et al., 2015; Ding et al., 2016; Kanitthamniyom and Zhang, 2018; Ryu et al., 2018; Lee et al., 2019).

Originally inspired by the ability of mussels to securely adhere to multiple surfaces in harsh environmental conditions, PDAM-based coatings have been applied to polymers to improve their haemocompatibility (Yang et al., 2012; Ye et al., 2015; Li et al., 2020; Tan et al., 2021). Microfluidic channel surfaces have been functionalised with PDAM through self-polymerising methods in closed channels (Shen et al., 2015; Kanitthamniyom and Zhang, 2018; Khetani et al., 2020; Park et al., 2022). The current overriding application of PDAM is as a spacer to create a very secure and simple anchor point for other molecules (Luo et al., 2013), including bioactive anticoagulants, such as heparin (Leung et al., 2015).

#### 3.3.4 Zwitterionic polymers

Zwitterionic polymers have both positive and negative charges, from cation and anion-containing groups respectively, while the overall charge is neutral (Sin et al., 2014; Blackman et al., 2019). The incentive to apply these polymers as a biocompatible surface layer comes from the similar dual-charge of the phospholipid membrane of mammalian cells (Schlenoff, 2014). Zwitterionic groups—Most commonly sulfobetaine, carboxybetaine, phosphobetaine and phosphorylcholine—Can be integrated to polymers referred to as polybetaines or polyzwitterionic materials (Chen et al., 2010; Schlenoff, 2014; Sin et al., 2014; Blackman et al., 2019). These polymers can be grafted to material surfaces and exert anti-fouling properties, increasing the surface hydrophilicity and forming a hydrated boundary layer—The same phenomenon which occurs at PEGylated surfaces (Chen et al., 2010; Schlenoff, 2014). Grafting of zwitterionic polymers on PDMS surfaces has exhibited improved haemocompatible properties: reducing fibrinogen adsorption, platelet deposition and haemolysis (Leigh et al., 2019; Kim et al., 2020; Jensen et al., 2021; McVerry et al., 2022; Mercader et al., 2022). Furthermore, grafting has been achieved on the inner surfaces of microfluidic channels (Plegue et al., 2018; Mercader et al., 2022) which suggests their applicability as a coating in microfluidic blood-contacting devices.

### 3.4 Commercial coatings

There are various blood-protective coatings commercially available, developed by medical device companies for use in coating of their medical devices such as cardiopulmonary bypass circuits (CPB). These commercial coatings, their mechanism of action (as biopassive, bioactive or combination), the molecular components, and the current applications are summarised in Table 2.

**TABLE 2:** Commercial blood-protective coatings, their mechanism of action and the molecular components employed to achieve this, and the devices on which they are currently applied.

Company	Commercial name	Mechanism of action	Coating component	Current applications	References
Medtronic	Balance™ Biosurface	Combination	Polyethylene oxide (PEO); sulphate and sulphonate groups	CPB circuits	Teligui et al. (2014), Willers et al. (2021)
	Trillium^®^ Biosurface	Combination	Heparin; PEO; sulphate and sulphonate groups	CPB circuits	Biran and Pond (2017), Hong and Waterhouse (2023)
Terumo	Xcoating™	Biopassive	Poly(2-methoxyethylacrylate) (PMEA)	CPB circuits, haemodialysers, catheters	Biran and Pond (2017)
Maquet Getinge group	BIOLINE	Bioactive	Heparin; albumin	CPB circuits, vascular graft	Hoshia et al. (2013), Biran and Pond (2017)
	SAFELINE	Biopassive	Albumin	CPB circuits	Preston et al. (2010), Zhang et al. (2021)
	SOFTLINE	Biopassive	Glycerol-poly(ethylene glycol)-ricinoleate	CPB circuits	Zhang et al. (2021)
LivaNova	PHISIO	Biopassive	Phosphorylcholine	CPB circuits, oxygenators	De Somer et al. (2000), Hei et al. (2023)

The coatings from Medtronic (*Balance™ Biosurface* & *Trillium® Biosurface*) differ only in the presence of heparin. They both consist of a primer layer on which is fixed polyethylene oxide (PEO) chains which deems the surface hydrophilic. The addition of sulphonate and sulphate groups contributes a negative-charge to repel platelets and also binds antithrombin to reduce thrombin production. Heparin, present only on the *Trillium® Biosurface* coating, increases the anti-coagulant activity (Medtronic, 2020).

The Maquet Getinge group uses three coatings: *SAFELINE*, *SOFTLINE*, and *BIOLINE*. The SAFELINE and SOFTLINE coatings do not contain heparin and their mechanism of operation is passivation. For SOFTLINE, this is particularly unique, utilising the hydrophilic polymer glycerol-poly(ethylene glycol)-ricinoleate with hydrophilic polymers. SAFELINE and BIOLINE are albumin-based, with BIOLINE being the bioactive option combining the addition of heparin (Getinge, 2018).

*Xcoating™* and *PHISIO*, from the companies Terumo and LivaNova respectively, are both heparin-free coatings, of which only the hydrophilic nature prevents blood coagulation. However, they achieve this with two different polymers: PMEA poly(2-methoxyethylacrylate) for *Xcoating™* and phosphorylcholine for PHISIO. Both of which reduce protein denaturation and platelet adhesion (Terumo, 2018; LivaNova, 2023).

### 3.5 Future prospects for haemocompatible coatings of microchannels

#### 3.5.1 Endothelialisation

The future direction of haemocompatible coatings in microfluidic channels is following a biomimetic approach. One highly anticipated method is lining channels with endothelial cells. Within the body, the inner surface of the vascular network is lined with endothelial cells to form an inherently haemocompatible monolayer (Neubauer and Zieger, 2021). Vascular endothelial cells regulate blood haemostasis through multiple anticoagulant, antithrombotic and procoagulant mechanisms. The biologically-relevant dimensions of microfluidic channels places them as a potential candidate for endothelialisation (Hesh et al., 2019).

The aspiration is such that the blood will not recognise a change in environment from exiting the vessels of the body to the lined channels of the device (Hesh et al., 2019). The use of patient-derived cells to create a personalised device would reduce the risk of adverse reactions and further improve haemocompatibility. However major challenges face this approach. Since endothelial cells are very reactive to shear stress and hydrodynamic conditions the operational flow conditions must be closely controlled to avoid cellular detachment or disturbing the integrity of the monolayer (Roux et al., 2020). Furthermore, the attachment surface must be prepared for long-term cell culture (Jana, 2019). Depending on the substrate material it will require some kind of base coating to encourage cell adhesion and to support the culture. Maintenance of the culture is also an important consideration. Controlled removal and renewal of dead cells is required to ensure an operational and complete coating. The regulatory pathway to bring a cell-based coating to market will be challenging. Some characteristics of endothelium anti-coagulant activity has inspired research into new approaches for haemocompatible coatings. Although some microfluidic networks have been endothelialised (Burgess et al., 2009; Hellmann et al., 2020; Lachaux et al., 2021) significant progress is required before it is a feasible clinical option.

#### 3.5.2 NO-releasing

Nitric Oxide (NO) is a soluble gas continuously produced and released into the blood by endothelial cells to regulate platelet activity, reducing their adhesion and aggregation to play a crucial anti-thrombotic role (Radomski et al., 1987; Emerson et al., 1999; Tousoulis et al., 2011; Porrini et al., 2020; Neubauer and Zieger, 2021). NO also modulates the inflammatory response (Kobayashi, 2010), T-cell mediated immunity (Bogdan, 2015; García-Ortiz and Serrador, 2018) and exhibits antibiotic properties (Wang et al., 2002). This response has been replicated in NO-releasing materials (Wu Y. et al., 2007b; Wu et al., 2007a; Seabra et al., 2010; Yang et al., 2015; Liu et al., 2017; Simon-Walker et al., 2017; Luo et al., 2018; Qiu et al., 2019; Sadrearhami et al., 2019; Devine et al., 2020; Lyu et al., 2020; Hosseinnejad et al., 2021; Mondal et al., 2021). Although their application to microchannels is limited so far, such coatings have been investigated for other biomedical applications, the use of polymers as base substrate means they are still relevant in the development of microfluidic devices. These approaches generally operate through the inclusion of a NO donor integrated into the coating matrix or immobilised on the material surface, the most common being S-Nitroso-N-acetylpenicillamine (SNAP) (Liu et al., 2017; Devine et al., 2020; Mondal et al., 2021), S-nitrosothiols (RSNOs) (Yang et al., 2015; Luo et al., 2018; Qiu et al., 2019; Hosseinnejad et al., 2021) or N-diazeniumdiolate precursors of NO (Wu B. et al., 2007a; Sadrearhami et al., 2019). Immobilisation can be achieved by grafting to protein spacers, as previously discussed (Liu et al., 2017). In attempts to further replicate the antithrombotic properties of the endothelium, these methods are often integrated with immobilised heparin (Wu B. et al., 2007a; Simon-Walker et al., 2017; Devine et al., 2020), which takes the place of endothelial heparan sulphate. However, the ability to control the release of NO over time is limited by the fact the molecule is highly reactive with a very short half-life. In the blood this is within the range of seconds (Weller, 2009; Fleming et al., 2017). This is also the case with the release of NO from N-diazeniumdiolate, where the half-life is only a few minutes, introducing technical difficulties with its manipulation (Jeong et al., 2018).

In an attempt to overcome this unstable release there is the alternative approach of introducing NO, instead in gaseous form. This is particularly relevant for microfluidics due to the efficient gas exchange capabilities in these devices (Thompson et al., 2020). The inclusion of NO in the sweep gas blend in oxygenators has previously gained interest in clinical settings, particularly in patients with severe respiratory conditions where there is some evidence that it improves oxygenation capabilities and reduces platelet activation (Mellgren et al., 1996; Keh et al., 1999; Skogby et al., 2003). However, more recent studies have called these results into question, revealing negligible effects or minimal improvements to blood condition after the addition of NO to the sweep gas during ECMO (Adhikari et al., 2007; Rossidis et al., 2020; Niebler et al., 2021; Fallon et al., 2022). Furthermore these therapies require further definition regarding the release flux of NO into the blood [the *in vivo* range for vascular endothelium is 0.5–4 × 10^−10^ mol cm^−2^ min ^−1^ (Vaughn et al., 1998)], since excessive levels can lead to detrimental effects including heart failure, blood pressure changes and neural defects (Khazan and Hdayati, 2014).

## 4 Discussion

Coatings are a critical part of improving haemocompatibility of blood-contacting devices. Since the emergence of polymeric microfluidic devices as new platforms for manipulating blood under predictable hydraulic conditions, the application of coatings to these devices must be considered. Until now the focus has been primarily on the mechanical experience of the blood and blood-processing functionalities, with the material interactions remaining an afterthought. Although it is true that haemodynamic forces contribute hugely to damage experienced by blood in microfluidic networks, and the methods of protective design have been previously reviewed (Szydzik et al., 2020; Astor and Borenstein, 2022; Feaugas et al., 2023), devices will not achieve acceptable haemocompatibility without appropriate coating of microchannel surfaces. This review provides a foundational understanding of the significance of material-blood interactions in microfluidic channels and the formation of thrombi, and how coatings can improve haemocompatibility.

Since their conception, materials and fabrication of microfluidic devices have represented a major bottleneck to their commercialisation (Roy et al., 2011; Temiz et al., 2015; Nielsen et al., 2020). In the context of blood-contacting microfluidics this is even more pertinent since the most common materials—Polymers hailed for their improved fabrication qualities—Promote thrombogenic pathways. However, as noted in the lack of publications and data in Table 1, haemocompatibility is not a priority during testing with the focus being biocompatibility instead. The two characteristics are not interchangeable, they have very different parameters and requirements. It is clear that there is more research needed to verify the haemocompatibility of these polymers—Not only for microfluidics but for blood-contacting medical devices in general.

The multi-step fabrication processes of microfluidic devices require pre-treatment or surface modification steps which challenge the application or introduction of coatings. This represents a major limitation to the methods by which coatings can be applied to the microchannels, and must be considered before selecting the coating. From a fabrication perspective, it is more simple to apply the coating after sealing the microchannels in order to preserve bonding integrity. However, for this to work the coating must be fluidic and applicable through the inlet and outlet ports.

In general, haemocompatible coatings take inspiration from the endothelium. Heparin remains the gold standard for clinical blood protective coatings, positioning it as the first port-of-call for coating microfluidic channels too. However, trends in research are moving away from this coating method and towards “heparin-free” options which, as discussed, are showing promising results.

Microfluidic devices offer new platforms where diffusion gradients can be closely controlled. This has incentivised their application to blood processing medical devices, such as oxygenators or dialysers. The most common microfluidic materials are polymers, such as PDMS, which are poorly suited to this application since their surfaces promote thrombogenic processes. Furthermore, the geometry of microchannels is such that the surface area to volume ratio is shifted: there is a high contact between the flowing fluid and material walls. It is therefore critical that the surface be altered to protect the blood and improve device haemocompatibilty.

Coatings applied to blood-contacting medical devices have been shown to greatly reduce blood trauma during their use and is standard clinical practice. However, the process of coating microfluidic channels is not without challenges. The coating application method is limited by the fabrication and assembly process, or *vice versa*. In addition, the shear forces exerted by fluids on microchannel surfaces can challenge the integrity of the coating and it must be ensured to withstand the dynamic environment. Plasma cleaning is a crucial step in the fabrication of microfluidic devices, mostly in order to activate the surface for bonding but critically to remove debris or impurities. Activation of the material surface by plasma is often also the first step in the coating process by freeing functional groups for bonding with the coating molecules.

Heparin is considered the gold standard in anti-coagulant coatings, it is a bioactive molecule which is often immobilised on surfaces via polymers or protein anchors. One such protein is albumin. This readily available protein can be adsorbed rapidly to hydrophobic polymer surfaces to block unwanted adsorption of plasma proteins, however it is displaced over time. A major method of biopassive coatings is the grafting of polymers. The most common being PEG-based, hydrophilic polymers which create a hydrated layer at the surface which reduces blood-material interactions. Polymer brushes, including PDAM, can also function as spacers to attach molecules—Such as heparin—To material surfaces to render them haemocompatible. Zwitterionic polymer coatings mimic the dual-charge phenomenon of the cell membrane to repel protein and cells from surfaces.

The ideal coating would of course be the endothelial layer itself. Some progress has been made to coat networks of microchannels with cells but further research is required to support this approach in clinical devices of the future. Recently, haemocompatible coatings have employed other biomimetic approaches which replicate the activity of the endothelium, one focus being NO-releasing coatings. It is clear that future haemocompatible coating developments will integrate protective features of the natural endothelium in order to provide a truly biomimetic, inert surface for interaction with blood.

The evolution of microfluidics technology towards high volume blood processing involves new considerations and challenges to achieve surface haemocompatibility.
