# Corrigendum: Challenge of material haemocompatibility for microfluidic blood-contacting applications

**DOI:** 10.3389/fbioe.2023.1297000

**Published:** 2023-10-10

**Authors:** Gwenyth Newman, Audrey Leclerc, William Arditi, Silvia Tea Calzuola, Thomas Feaugas, Emmanuel Roy, Cécile M. Perrault, Constance Porrini, Mikhael Bechelany

**Affiliations:** ^1^ Department of Medicine and Surgery, Università degli Studi di Milano-Bicocca, Milan, Italy; ^2^ Eden Tech, Paris, France; ^3^ Institut Européen des Membranes, IEM, UMR 5635, Univ Montpellier, ENSCM, Centre National de la Recherche Scientifique (CNRS), Place Eugène Bataillon, Montpellier, France; ^4^ École Nationale Supérieure des Ingénieurs en Arts Chimiques et Technologiques, Université de Toulouse, Toulouse, France; ^5^ Centrale Supélec, Gif-sur-Yvette, France; ^6^ UMR7648—LadHyx, Ecole Polytechnique, Palaiseau, France; ^7^ Gulf University for Science and Technology (GUST), Mubarak Al-Abdullah, Kuwait

**Keywords:** haemocompatibility, microfluidics, surface modification, coating, medical devices, thrombosis, biomaterials

In the published article, there was an error in the **Author** list, and author “Cécile M. Perrault” was erroneously excluded. The corrected author list appears below.

“Gwenyth Newman^1,2^*, Audrey Leclerc^3,4^, William Arditi^2,5^, Silvia Tea Calzuola^2,6^, Thomas Feaugas^1,2^, Emmanuel Roy^2^, Cécile M. Perrault^2^, Constance Porrini^2†^ and Mikhael Bechelany^3,7†^.”

The corrected **Author contributions** section appears below.

“GN conceived and wrote the manuscript. AL contributed in writing the manuscript. WA, TF, SC, and ER contributed in reviewing the manuscript. CP, WA, CMP, and MB reviewed and structured the manuscript, and managed the project. All authors contributed to the article and approved the submitted version.”

The corrected **Conflict of interest** statement appears below.

“Authors GN, WA, SC, TF, ER, CMP, and CP were employed by the company Eden Tech.

The remaining authors declare that the research was conducted in the absence of any commercial or financial relationships that could be construed as a potential conflict of interest.”

The authors apologize for this error and state that this does not change the scientific conclusions of the article in any way. The original article has been updated.

